# Acute Exercise Facilitates the N450 Inhibition Marker and P3 Attention Marker during Stroop Test in Young and Older Adults

**DOI:** 10.3390/jcm7110391

**Published:** 2018-10-26

**Authors:** Shu-Shih Hsieh, Chung-Ju Huang, Chien-Ting Wu, Yu-Kai Chang, Tsung-Min Hung

**Affiliations:** 1 Department of Physical Education, National Taiwan Normal University, Taipei 106, Taiwan; stonehsieh79218@gmail.com; 2 Graduate Institute of Sports Pedagogy, University of Taipei, Taipei 106, Taiwan; crhwang@utaipei.edu.tw; 3 Department of Exercise and Sport Science, University of South Carolina Upstate, 800 University Way, Spartanburg, SC 29303, USA; showpan2004@gmail.com; 4 Department of Physical Education & Institute for Research Excellence in Learning Science, National Taiwan Normal University, Taipei 106, Taiwan

**Keywords:** aerobic exercise, cognitive inhibition, Stroop test, N450, P3

## Abstract

While considerable evidence supporting the positive influence of acute exercise on cognitive inhibition, little is known regarding the underlying cognitive processes. There is also little neuroelectric evidence regarding the effects on older adults of acute exercise-elicited cognitive benefits. Thus, our objective was to explore the possible neural markers underlying improved cognitive inhibition, with particular attention to the N450 and P3 components, following acute exercise. Another aim was to investigate whether cognitive gains seen in young adults are replicated in older adults. Twenty-four young males and 20 older males underwent either a single bout of aerobic exercise or video-watching in counterbalanced order. Afterwards, cognitive inhibition was assessed by the Stroop test. Results revealed that acute exercise resulted in shorter response time regardless of age or congruency. Regarding the neuroeletric data, acute exercise resulted in larger P3 amplitude and smaller N450 amplitude regardless of congruency or age. Further, following exercise, changes in response time interference were correlated with changes in incongruent N450 amplitude. Collectively, acute exercise-facilitated conflict monitoring and attention control, as signified by the N450 and P3 components, may be the underlying processes leading to better Stroop performance, with conflict monitoring having a stronger association with task performance. Further, cognitive gains resulting from acute exercise were found to the same extent in both young and older adults.

## 1. Introduction

Acute exercise has been related to improvement in executive function [1,2], particularly the inhibition process [2] referring to the suppression of attention at the perceptual, cognitive, and motor level [3]. Inhibition at the cognitive level (i.e., cognitive inhibition), in particular, refers to the overriding of prepotent mental representations [4] or the resisting of proactive interference from previously acquired information [5]. This cognitive domain is modulated by the dorsolateral prefrontal cortex (PFC), a brain area whose functioning is known to be particularly affected by exercise [6]. Moreover, it has been linked to health- and social-related behaviors such as addiction [7,8], obesity [9], and school [10,11] or job success [12]. For these reasons, inhibition has been a major focus in the literature on the relationship between acute exercise and executive function. Accumulating evidence supports the positive influence of acute continuous aerobic exercise (CAE) on cognitive inhibition as assessed by the Stroop color-word test, a paradigm that specifically aims to assess this cognitive domain [13,14,15,16,17]. As such, studies have found a shorter response time (RT) [13,17] or higher response accuracy [15] in both the congruent and incongruent trials following CAE, suggesting a generalized CAE-elicited effect. On this basis, previous research has postulated that acute CAE may result in improved inhibition of task-irrelevant information. However, one limitation of behavioral data is the lack of information on specific cognitive processes underlying better inhibition performance given specific inhibitory processes may occur within a narrow window of time.

While cognitive inhibition may result from many different cognitive processes, conflict monitoring, the process of monitoring performance for simultaneously competing response options [18], may be particularly important. If conflict is not adequately detected, and subsequent adjustment in behavior and neural resources are not implemented, goal-directed behaviors will be compromised [18]. Event-related brain potentials (ERP) enable real-time measurement of changes in electric activity relating to distinct cognitive operations (e.g., stimulus encoding, stimulus processing, response preparation, response selection) [19], with a temporal resolution down to milliseconds (ms), which enables detection of specific inhibitory processes. Moreover, the N450 component from ERP is particularly well-suited to understand conflict monitoring.

N450, also known as medial frontal negativity, is a frontocentral-distributed and stimulus-locked slow wave, with an onset of 400–600 ms after target presentation [18]. N450 represents an index of conflict that is elicited by tasks such as the Stroop test, and likely reflects activity of neural generators localized in the dorsal anterior cingulate cortex (ACC) [20], a brain area responsible for detection of conflict in performance or environment, as well as subsequent behavioral adjustments [20]. During the Stroop test, N450 amplitude tends to be larger (i.e., more negative) in the incongruent compared to the congruent trials, suggesting increased neural resources being used in the detection of competing response options [21] or interference [22]. Since enhanced inhibitory processing is assumed to be one of the cognitive changes resulting in improved Stroop performance following acute exercise, investigation of N450 should be of particular interest in this line of research. However, only one study has investigated this topic to date. Chang et al. [13] found that college students had smaller N450 amplitude (i.e., more positive) and shorter N450 latency following acute CAE. This exercise-elicited effect was observed in both congruent and incongruent trials, suggesting a more effortless state of conflict detection regardless of task difficulty. However, the Chang et al. study only focused on young adults and so it is not clear whether these results generalize to other populations, in particular older adults.

N450 is affected by the aging process. It has been reported that during the Stroop test, older adults display larger N450 amplitude or longer N450 latency [23], suggesting more neural resources and prolonged processing time are required during the detection of task-induced conflict and subsequent signaling of behavioral adjustments. This deterioration in conflict detection could be accounted for by a lower neural availability of the PFC and an over-activation of the ACC [24], resulting in an inefficient state of conflict detection and/or subsequent behavioral adjustments. This age-related decline is associated with worse task performance [23]. Since declines in inhibition have been related to greater time required to implement correct responses [25] or frequent forgetting in the presence of competing mental concepts [26], investigation of possible factors that may lead to improvement, such as exercise, is desirable. Given that the cortical area mostly closely related to N450, the ACC, is affected by aerobic exercise in older adults [27,28], it is reasonable to suppose that aerobic exercise may also modulate N450.

The P3 component, on the other hand, has been consistently associated with improved inhibition following acute exercise. P3, also known as P3b or target-P3, is an ERP elicited between 300–700 ms after stimulus onset, and is typically maximal over the centroparietal region [29]. P3 amplitude reflects the amount of attention allocated during the processing of target stimuli, and P3 latency reflects stimulus processing speed [29]. A growing body of evidence has found that acute CAE results in increased P3 amplitude using the Stroop test [13,17] and other inhibition-related tasks (e.g., Flanker task) [30,31,32]. This exercise-elicited effect has been observed in both congruent and incongruent trials, suggesting better attention engagement regardless of task demands. However, as in the case of N450, there is little evidence about the effects of acute CAE on P3 in older adults, who are also reported as having smaller P3 amplitude [33] and delayed P3 latency [33,34] during the Stroop test, suggesting a reduced ability to invest sufficient attention resources during stimulus processing or slower stimulus processing speed [29]. These functional declines in P3 in older adults could be accounted for by deteriorations in the locus coeruleus circuit (LC) and norepinephrine (NE) system [35], with deteriorated phasic response of the LC circuit and decreased availability of NE in the PFC resulting in a delayed onset and smaller/less evident (more flat) amplitude of the P3 [36]. Given that the brain areas associated with P3 (e.g., prefrontal, temporal, and parietal cortices) are regulated by aerobic exercise in older adults [27,28,37], it is worth examining whether P3 could itself be modulated by CAE in older adults.

The primary objective of the current study was to explore the cognitive processes underlying improved cognitive inhibition following acute exercise, with particular focus on the N450 and P3 components which reflect conflict monitoring and attention engagement process, respectively. A secondary aim of this study was to investigate whether finding reported in relation to N450 and P3 in young adults can be generalized to older adults. Based on previous findings, it was hypothesized that acute CAE would lead to shorter RT, higher response accuracy, smaller N450 amplitude, and larger P3 amplitude regardless of task difficulty in young adults. The same pattern of effects was also expected to be found in older adults. Latency measure was not collected in this study because of concerns that this measure may not be meaningful in older adults given its prolonged time course and relatively flat waveform. To date, despite the fact that research has supported a role of acute exercise in facilitating cognitive function, we have little evidence of the neuroelectric underpinnings, particularly in older adults. Our findings may have implications for tailoring exercise interventions to improve inhibition performance and neural functioning in young and older adults.

## 2. Methods

### 2.1. Participants

The current study recruited 24 young males (Mean_age_ = 24.0 ± 3.1 years) and 20 older males (Mean_age_ = 70.0 ± 3.3 years). Only men were recruited because prior data has suggested the presence of sex-related differences in secretion of hormones relating to arousal [38], metabolic state [39], or cognitive function [38] (e.g., dehydroepiandrosterone sulfate, DHEA-S) following acute CAE, with women showing greater exercise-induced secretions [40]. It is possible that men and women have different cognitive arousal patterns or metabolic changes following exercise, which may, in turn, confound the effects of acute exercise. This sample size was determined based on an a priori power analysis using G*Power 3.4 software (alpha = 0.05, power = 0.80) to detect the effects of acute CAE on Stroop performance (partial eta square effect size of 0.16) [17].

Participants in the current study were limited to those who reported that they: (1) Right-handed; (2) were free from any of the medical conditions listed on the Physical Activity Readiness Questionnaire (PARQ); (3) were free from cardiovascular, cerebrovascular, or neurological disorders; (4) had normal or corrected-to-normal vision; (5) had at least 12 years of education; and (6) were free from cognitive impairment or mental disorders as indicated by obtaining a score of >24 points on the Mini Mental State Examination (MMSE) or a score between 0 to 13 points on the Beck Depression Inventory (BDI). Written informed consent was obtained from all participants via a form approved by the Institutional Review Board of National Taiwan Normal University.

### 2.2. Measurements

#### 2.2.1. Demographic and Anthropometric Measures

Age, years of education, and socioeconomic status (measured on a 5-point scale with ‘5’ representing the highest status) were assessed by self-report. Height and weight were measured in the laboratory. In addition, body mass index (BMI) was calculated as weight (kg)/height (cm^2^). General cognitive ability was assessed by the digit span forward, digit span backward, and digit sequence span tests from the fourth edition of the Wechsler Adult Intelligence Scale (WAIS-IV). Raw scores from each subtest were combined and converted into an age-adjusted standard score (ranging from 2 to 19 points). This measure has been widely employed as a proxy measure of general cognitive ability [7,11]. Physical activity levels were assessed by the Chinese version of International Physical Activity Questionnaire (IPAQ) [41].

#### 2.2.2. Exercise Manipulation Measures

Heart rate (HR) has been found to be a valid indicator of physiological arousal and used as a measure of exercise intensity in the acute exercise-cognition literature [13,17]. Data was collected using a polar HR monitor (Polar RS800CX; Polar ElectroOy, Kempele, Finland) at the following time points: (1) HR baseline: HR measured during a 5-min rest before treatment; (2) HR treatment: HR measured during the exercise or video treatment; (3) HR post: HR data taken during a 15-min post-treatment rest; and (4) HR task: HR measures taken during the Stroop test.

The rating of perceived exertion (RPE) scale, which ranges from 6 to 20, and was used to provide a subjective rating of individuals’ perceptions of their efforts during exercise [42]. RPEs were recorded every 4 min during a 20-min bout of CAE.

#### 2.2.3. Cardiorespiratory Fitness Testing

Cardiorespiratory fitness (CRF) levels were determined using the single-stage submaximal treadmill walking test (SSTWT) developed by Ebbeling et al. [43], a commonly used protocol for predicting maximal oxygen consumption (VO_2peak_) in adults know to have prediction coefficients in the range of r = 0.86–0.96. HR was collected during testing, in two 4-min stages. During the first stage, participants were instructed to warm up on a treadmill at a self-paced speed of between 3.0 to 4.5 mph (4.8 to 7.2 kmh) at a 0% gradient. The speed was increased until participants raised their HR to 60–70% of age-predicted HR_max_ (calculated using the formula 206.9 − (0.67 × Age)). In the second stage, participants maintained the speed they had established in the warm up stage while the gradient was raised to 5%. Steady-state HR (SSHR) was determined during the last 2 min of the second stage. The predicted value of VO_2peak_ was determined following the formula for males developed by Ebbeling et al. [43]: VO_2peak_ (mL/kg/min) = (15.1 + (21.8 × Final speed) − (0.327 × SSHR) − (0.263 × Final speed × Age) + (0.00504 × SSHR × Age) + 5.98). Individual VO_2peak_ measures were transformed into age-adjusted percentiles (VO_2peak_ percent rank) using norms from the American College of Sports Medicine (ACSM) [44].

#### 2.2.4. Modified Stroop Color-Word Test

The modified Stroop color-word test, adapted from previous studies [13,14,15,16,17], was used to measure cognitive inhibition. The task was programmed using STIM 2.0 software (Neuroscan Ltd, El Paso, TX, USA). This task consisted of two types of trials: congruent and incongruent. Congruent trials used one of three color words [i.e., “綠” (GREEN), “紅” (RED), “黃” (YELLOW)] printed in the Chinese language in the corresponding ink color [e.g., 紅 (RED) printed in red]. The incongruent trials consisted of the same three words, but printed in non-corresponding colors [e.g., 綠 (GREEN) printed in red]. The stimuli were 2 × 2 cm and were displayed focally on a 17-inch computer monitor that was placed 75 cm in front of the participants, with a visual angle of 2°. Each trial began with the target stimuli being presented for 500 ms. Participants were instructed to indicate the color of the presented stimuli as fast as possible within a 1500-ms response window by pressing keys with their dominant hand as follows: “G” for red, “H” for yellow, and “J” for green. The inter-trial interval is 500 ms. Each block comprised of 60 trials, consisting of 42 congruent and 18 incongruent trials, displayed randomly. Six blocks of trials were completed with a total of 360 trials.

Before the beginning of formal testing, a neutral block with 42 trials was performed for the purpose of familiarization with color allocation. The stimuli in the neutral block were rectangles printed in the three colors. Afterwards, two practice blocks with a total of 42 trials were performed until participants reported being able to understand the entire task procedure, and were able to reach 90% and 85% response accuracy for young and older adults respectively. These age-specific criteria were derived from pilot data. In calculating performance indices, trials with no response, an incorrect response, or reaction times (RT) either less than 200 ms or longer than 1500 ms were discarded. Mean RT of correct trials (ms) and mean response accuracy of all trials (%) were calculated. In addition, interference scores in mean accuracy (i.e., accuracy in congruent trials − accuracy in incongruent trials) and mean RT (i.e., RT in incongruent trials − RT in congruent trials) were calculated to represent the Stroop interference (SI) effect as supplementary indices.

#### 2.2.5. Electroencephalographic Recordings

Electroencephalographic (EEG) activity was recorded with 64 electrode sites using an elastic electrode cap (Quick-Cap, Compumedics Neuroscan, Inc., Charlotte, NC, USA) according to the modified International 10–20 System. Continuous data was referenced online using a midline electrode between CZ and CPZ, with AFZ electrode serving as the ground. The electrooculographic (EOG) activity was measured by using four electrodes placed at the outer canthus of each eye, and above and below the left orbit. All electrodes maintained impedances <10 kΩ before data recordings. The continuous data acquisition was performed with a sampling rate of 1000 Hz, a DC- to 200-Hz filter, and a 60-Hz notch filter using a Neuroscan SynAmps2 amplifier.

For data reduction, continuous data was firstly re-referenced to averaged mastoids (M1, M2). The EEG data was filtered using a zero-phase shift of 0.1- to 30-Hz band-pass cutoff (12 dB/octave), and the EOG activity was corrected using the algorithm described by Semlitsch et al. [45]. Epochs were created from −200 to 1000 ms relative to stimulus onset, baseline-corrected using the mean amplitude of the 200-ms window before stimulus. Next, epochs with incorrect responses or an identified artifact which exceeded ±100 μV were discarded. The final numbers of epoch included into analyses were: congruent trials: young: 157.2 ± 50.2 and 174.6 ± 46.0 trials for young and older adults; incongruent trials: 70.8 ± 16.1 and 74.2 ± 15.5 trials for young and older adults.

**Figure 1:**
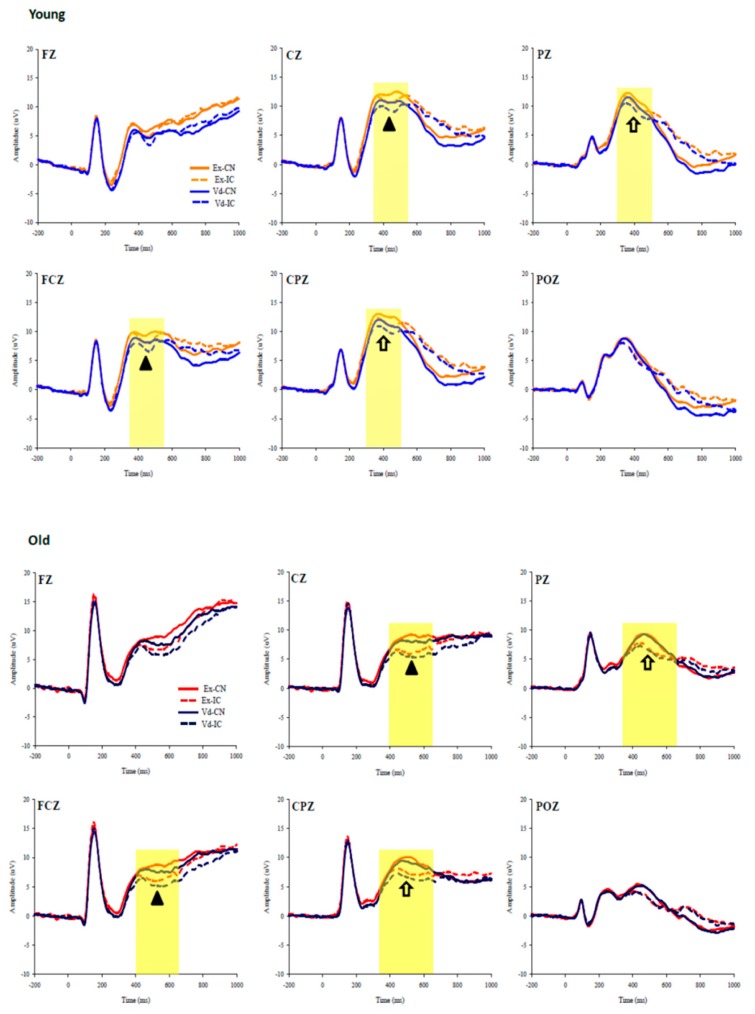
Grand-averaged waveforms at the midline electrodes (FZ, FCZ, CZ, CPZ, PZ, POZ) during the Stroop test in the exercise and the video-watching conditions. Upper and bottom panels represent data on young and older adults, respectively. Triangle (▲): The N450 component. Arrow (⇧): The P3 component. Ex-CN: Congruent trials in the exercise condition. Ex-IC: Incongruent trials in the exercise condition. Vd-CN: Congruent trials in the video-watching condition. Vd-IC: Incongruent trials in the video-watching condition.

Rather than calculating peak amplitude and peak latency for the N450 and P3 components, the current study calculated mean amplitude given that: (a) older adults had no evident peaks in either component, as depicted by the grand-averaged waveforms (Figure 1); (b) it could be problematic to use peak amplitudes when comparing young and older adults given that aging affects the onset and time course of N450 [23] and P3 [46]; and (c) calculating peak amplitude would be problematic considering the unequal number of congruent and incongruent trials in which the latter would have a lower signal-to-noise ratio [19], particularly for the N450 component. Clayson and colleagues [47] have validated the use of mean amplitude over peak amplitude or other adjusted methods, showing that the former provides robust ERP data regardless of changes in background artificial noise.

The N450 mean amplitude was derived by calculating the mean amplitude value within the 350- to 550-ms, and 400- to 650-ms time windows after stimulus onset for young and older adults, respectively. Likewise, the P3 mean amplitude was obtained by taking mean values within the 300- to 500-ms and 350- to 650-ms time windows after stimulus onset for young and older adults, respectively. These time windows were selected based on previous studies [19,46,48,49] and visual inspection of the grand-averaged waveforms (Figure 1).

### 2.3. Procedures

Each participant visited the laboratory on 2 occasions, separated by at least 96 h to avoid learning effects (average interval between condition: 10.0 ± 5.5 and 9.9 ± 4.1 days for young and older adults, respectively). Participants were asked arrive at the laboratory at the same time on both occasions. They were also required to refrain from caffeine and alcohol intake for 12 and 24 h respectively, avoid severe exercise for 12 h, as well as refrain from food consumption for one hour prior to each condition.

A counterbalance, crossover design was used for these two conditions. Half of the participants were assigned to the exercise condition in the first visit and the control condition in the second visit, while the other half were assigned to the control condition in the first visit and the exercise condition in the second visit. In the exercise condition, each participant first sat quietly in a sound-attenuated and magnetic-resistant chamber for 5 min after the elastic electrode cap was fitted to their head. Next, participants engaged in the familiarization and practice blocks of the Stroop test.

Afterwards, they warmed up for 5 min on a motor-driven treadmill, then performed a 20-min bout of moderate intensity aerobic exercise (defined as 60–70% of HR_reserve_). Target HR during exercise for each participant was pre-determined using the formula: ((HR_max_ − Resting HR) × 60–70%) + Resting HR [44], followed by a 5 min cool down period. This protocol was adapted from recent studies which demonstrated cognitive benefits of acute exercise in different age groups [2], and was consistent with the exercise guidelines for promoting health across the lifespan established by the ACSM [44]. After exercise, participants sat quietly in the sound-attenuated chamber for 15 min before being administered the Stroop test. This interval was obtained by referring to results from the meta-analytical review of Chang et al. [1] and other empirical studies [13,15,17].

The first half of the procedure in the control condition was identical to the exercise condition except that participants were instructed to sit quietly and watch a video relating to sport science in the same sound-attenuated chamber for 30 min. The use of a video-watching control condition was based on other relevant studies in the area [30,31,32,50]. In the latter half of the video condition, participants completed the demographic and fitness measures. The entire study protocol was approved by the ethics committee of National Taiwan Normal University and followed the ethical standards described in the Declaration of Helsinki.

### 2.4. Data Analysis

Statistical analyses were performed using the SPSS 22.0 software system, with a family-wise alpha of 0.05 set as the significance criteria. To test the homogeneity between groups, independent-samples *t*-tests were initially performed on all descriptive and fitness measures.

To examine the effects of exercise on HR, a 2 (Group: young, older) × 2 (Condition: exercise, video) × 4 (Time: baseline, treatment, post, task) repeated-measured analysis of variance (RM ANOVA) was utilized. An independent *t*-test was performed to test group differences in RPE during exercise.

For behavioral and neuroelectric indices, preliminary analyses were performed to test whether the observed experimental effects were due to the effect of condition order. As such, an additional between-subjects factor with two levels (Order: video→exercise, exercise→video) were included into the analyses described below. For behavioral indices, 2 (Group) × 2 (Condition) × 2 (Congruency: Congruent, incongruent) RM ANOVAs were performed on mean accuracy and mean RT. Secondary 2 (Group) × 2 (Condition) RM ANOVAs were performed on interference scores in accuracy and RT.

**Figure 2:**
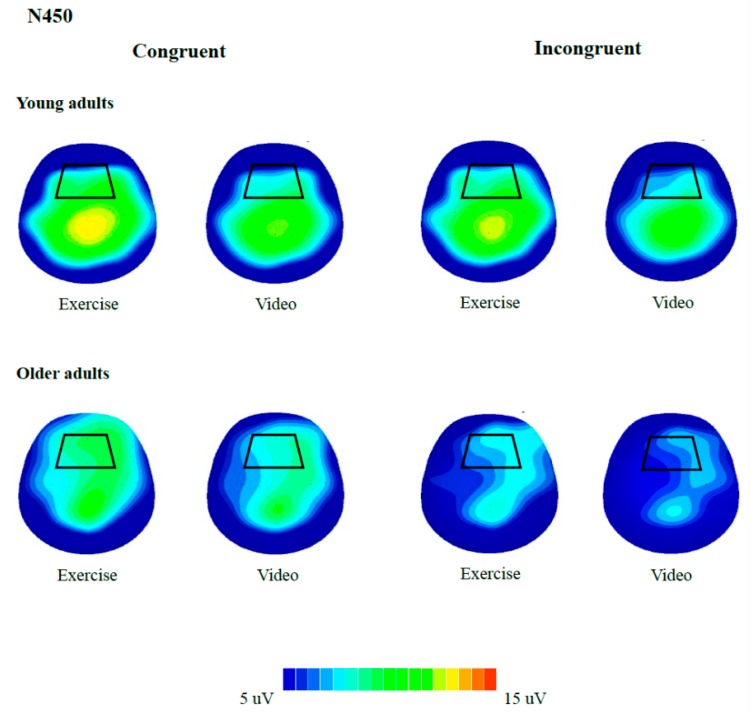
Topographic distributions of the N450 component in young (350–550 ms) and older adults (400–650 ms). Distribution (spectrum: Blue to red) is illustrated for the congruent and incongruent trials as modulated by exercise, with areas squared represent the frontocentral regions.

**Figure 3:**
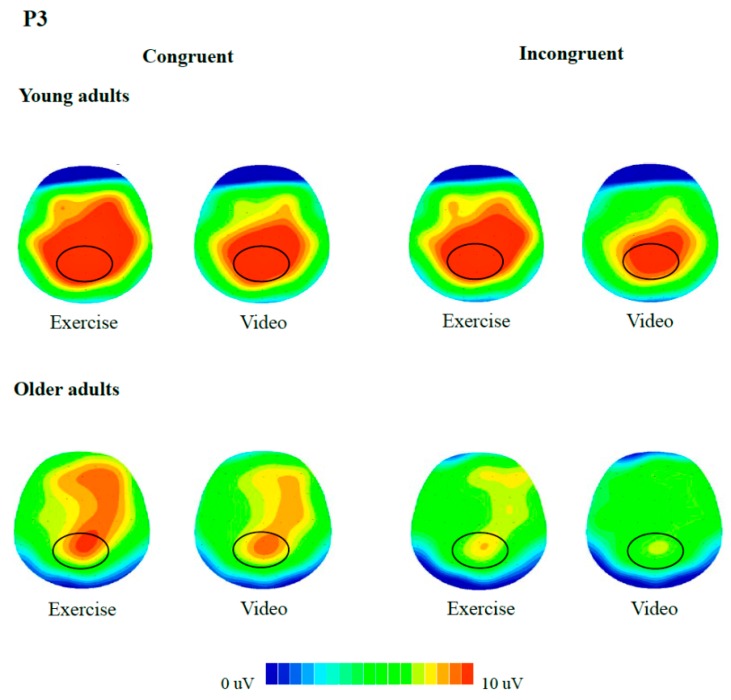
Topographic distributions of the P3 component in young (300–500 ms) and older adults (350–650 ms). Distribution (spectrum: Blue to red) is illustrated for congruent and incongruent trials, with or without exercise, with the centroparietal regions circled.

For neuroelectric indices, 2 (Group) × 2 (Condition) × 2 (Congruency) RM ANOVAs were performed on N450 and P3. Regions of interest were separately identified for the two components based on: (a) Previous studies which indicated that P3 was centroparietal distributed [51] while N450 is frontocentral distributed [48]; (b) the grand-averaged waveforms (Figure 1); and (c) voltage maps (Figure 2 and Figure 3). Recordings of N450 were collapsed across electrodes around the frontocentral region (i.e., FC1, FCZ, FC2, C1, CZ, C2) while data on P3 were averaged across electrodes around the centroparietal area (i.e., CP1, CPZ, CP2, P1, PZ, P2). Greenhouse-Geisser corrections were used when the assumption of sphericity was violated. Post hoc comparisons were corrected with Bonferroni-corrected *t*-tests. Partial eta square (η^2^_p_) effect sizes were reported in addition to the significance testing, with η^2^_p_ of 0.01, 0.06, 0.14 indicating small, medium, and large effect sizes, respectively [52].

To further investigate whether change in P3 or N450 accounted for change in task performance following CAE, Pearson bivariate correlations were performed across all 44 participants. Data of differences between CAE and video-watching on incongruent RT, incongruent response accuracy, RT interference, accuracy interference, incongruent P3 amplitude, and incongruent N450 amplitude were obtained by subtracting values in the video-watching condition from the exercise condition (exercise − video). 

## 3. Results

### 3.1. Demographic Data

Participants’ demographic and fitness data are summarized in Table 1. Independent-samples t-tests showed group difference in age, *t*(42) = −47.8, *p* < 0.001, VO_2peak_, *t*(42) = 9.9, *p* < 0.001, and raw scores in WAIS-IV, *t*(42) = 7.2, *p* < 0.001. Group differences in WAIS-IV and VO_2peak_ were no longer significant when standardized measures were compared (i.e., WAIS-IV standard score, VO_2peak_ percent rank) (*p*’s > 0.255). No group differences in other demographic variables were found (*p*’s > 0.087).

### 3.2. Exercise Manipulation Measures

**Figure 4:**
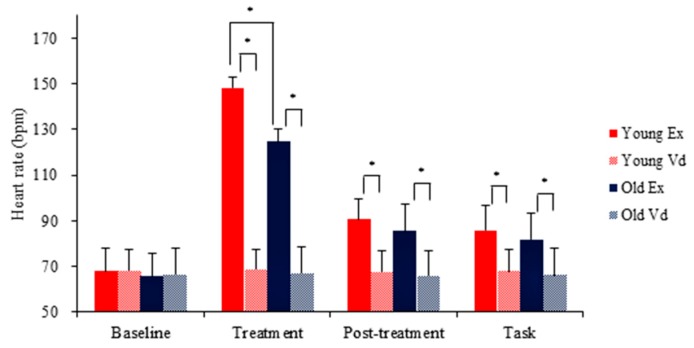
Fluctuations in heart rate (HR) as a function of Condition and Time. Young Ex: Exercise condition in young adults. Young Vd: Video-watching condition in young adults. Old Ex: Exercise condition in older adults. Old Vd: Bideo-watching condition in older adults. * *p* < 0.05.

With regard to HR, there was a main effect of Condition, *F*(1, 42) = 1108.2, *p* < 0.001, η^2^_p_ = 0.96, and Time, *F*(3, 40) = 1361.7, *p* < 0.001, η^2^_p_ = 0.97 and a Group × Condition × Time interaction, *F*(3, 40) = 28.4, *p* < 0.001, η^2^_p_ = 0.68. Decomposition of the interaction showed that HR was higher in the exercise condition relative to the video condition, post-treatment relative to both the rest and task time points in both age groups. Further, young adults had higher in-exercise HR than older adults. Figure 4 illustrates these HR fluctuations.

Mean RPE ratings during exercise were 11.7 ± 1.0 for young adults and 11.9 ± 1.0 for older adults. This ratings correspond to a “light” to “somewhat hard” level in the Borg’s scale (Borg, 1996), suggesting both groups exercised at a moderate level. Independent *t*-test showed no group difference in RPE during acute CAE.

### 3.3. Behavioral Performance

Preliminary analyses for condition order effect revealed Order × Condition interactions in mean accuracy, *F*(1, 40) = 4.7, *p* = 0.037, η^2^_p_ = 0.11, and mean RT, *F*(1, 40) = 19.1, *p* < 0.001, η^2^_p_ = 32. Accordingly, Order was included as a covariate into ANCOVAs on mean accuracy and mean RT, including *F* statistics and simple main effect testing involving Condition as factor.

After controlling for the order effect, the RM ANCOVA on mean accuracy showed a significant main effect of Congruency, *F*(1, 41) = 4.6, *p* = 0.038, η^2^_p_ = 0.10, with the congruent trials (98.3%) having a higher overall accuracy than incongruent trials (91.2%). However, neither the main effect of Group or Condition nor the interactions between factors were significant (*p*’s > 0.11). Secondary analysis on interference scores also found neither main effects nor interaction (*p*’s > 0.40).

Analyses on mean RT, after controlling for order effect, found main effects of Group, *F*(1, 41) = 78.2, *p* < 0.001, η^2^_p_ = 0.66, Condition, *F*(1, 41) = 25.2, *p* < 0.001, η^2^_p_ = 0.38, and Congruency, *F*(1, 41) = 24.2, *p* < 0.001, η^2^_p_ = 0.37. A Group × Congruency, *F*(1, 41) = 29.7, *p* < 0.001, η^2^_p_ = 0.42, and a Condition × Congruency interaction, *F*(1, 41) = 4.6, *p* = 0.037, η^2^_p_ = 0.10 were found.

Decomposition of the Group × Congruency interaction showed that young adults had shorter RT compared with older adults during both the congruent (Young: 431.5 ms; Older: 601.5 ms; *t*(42) = −8.9, *p* < 0.001) and incongruent trials (Young: 498.8 ms; Older: 765.4 ms; *t*(42) = −8.5, *p* < 0.001). RT was shorter during the congruent trials relative to incongruent trials both in young (Congruent: 431.5 ms; Incongruent: 498.8 ms; *t*(23) = −8.0, *p* < 0.001) and older adults (Congruent: 601.5 ms; Incongruent: 765.4 ms; *t*(19) = −9.9, *p* < 0.001).

**Figure 5:**
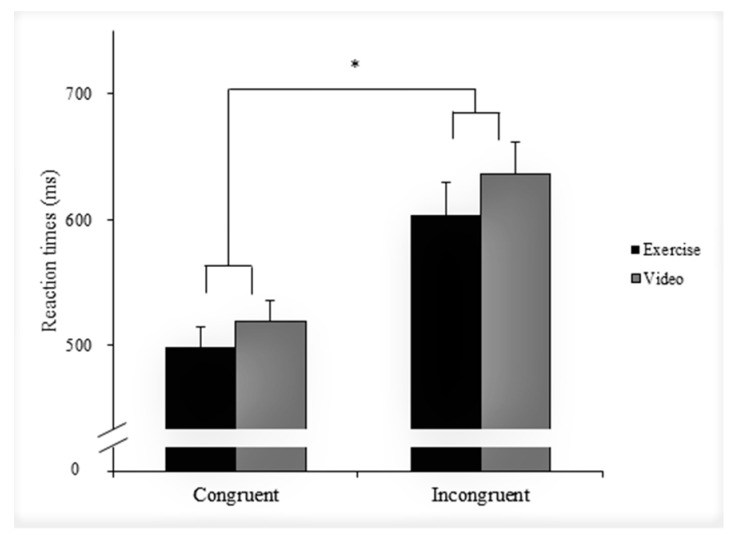
Mean (± 1 SE) reaction times during the Stroop test in the exercise (black bar) and video conditions (grey bar). Data were collapsed across young and older adults. * *p* < 0.05.

On the other hand, decomposition of the Condition × Congruency interaction showed that RT was shorter following exercise than following video-watching during the congruent (Exercise: 498.4 ms; Video: 519.1 ms; *F*(1, 42) = 29.7, η^2^_p_ = 0.37, *p* < 0.001) and incongruent trials (Exercise: 604.2 ms; Video: 635.8 ms; *F*(1, 42) = 21.6, η^2^_p_ = 0.34, *p* < 0.001). Results also indicated that RT was shorter during the congruent trials compared with that during incongruent trials in both the exercise (Congruent: 498.4 ms; Incongruent: 604.2 ms; F(1, 42) = 10.7, η^2^_p_ = 0.20, *p* = 0.002) and video condition (Congruent: 519.1 ms; Incongruent: 635.8 ms; *F*(1, 42) = 15.1, η^2^_p_ = 0.26, *p* < 0.001), with the former revealing a smaller congruency effect. Figure 5 depicts RT during the Stroop test in the exercise and video condition.

A follow-up two-way ANOVA analysis on RT interference score revealed a main effect of Group, *F*(1, 42) = 29.9, *p* < 0.001, η^2^_p_ = 0.42, with the older adults (163.9 ms) showing larger interference scores relative to young adults (67.3 ms). A main effect of Condition was also found, *F*(1, 42) = 5.0, *p* = 0.030, η^2^_p_ = 0.11, with smaller interference scores following exercise relative to following video-watching (Exercise: 110.0 ms; Video: 121.2 ms).

### 3.4. Neuroelectric Performance

With regards to ERPs, analyses on the P3 component showed a main effect of Condition, *F*(1, 42) = 7.5, *p* = 0.009, η^2^_p_ = 0.15, with larger amplitudes following exercise (9.3 μV) relative to video-watching (8.5 μV). Results also indicated a main effect of Congruency, *F*(1, 42) = 30.9, *p* < 0.001, η^2^_p_ = 0.42, with larger amplitudes during the congruent trials (9.5 μV) than that during incongruent trials (8.3 μV). A main effect of Group was observed as well, *F*(1, 42) = 7.1, *p* = 0.011, η^2^_p_ = 0.14, with the young adults (10.6 μV) having larger amplitudes than older adults (7.3 μV). There was no interaction between factors (*p*’s > 0.08).

As for the N450 component, there was a main effect of Condition, *F*(1, 42) = 8.9, *p* = 0.005, η^2^_p_ = 0.17, with larger amplitudes (less negative) following exercise (9.0 μV) compared with following video watching (7.9 μV). A main effect of Congruency was also observed, *F*(1, 42) = 24.7, *p* < 0.001, η^2^_p_ = 0.37, which was superseded by a Congruency × Group interaction, *F*(1, 42) = 4.8, *p* = 0.035, η^2^_p_ = 0.10. Decomposition of the interaction showed that while young adults (10.2 μV) had similar amplitudes to older adults (8.2 μV) during the congruent trials, they had larger amplitudes relative to older adults during the incongruent trials (Young: 9.4 μV; Older: 6.1 μV; *t*(42) = 2.1, *p* = 0.044). Results also showed a Congruency effect both in the young adults (Congruent: 10.2 μV; Incongruent: 9.4 μV; *t*(23) = 2.1, *p* = 0.046) and older adults (Congruent: 8.2 μV; Incongruent: 6.1 μV; *t*(19) = 4.7, *p* < 0.001). For details of the raw data and ANOVA/ANCOVA results related to behavioral measures and neuroelectric indices, please refer to Table 2 and Table 3.

### 3.5. Relations between Behavioral and ERP Measures

**Figure 6:**
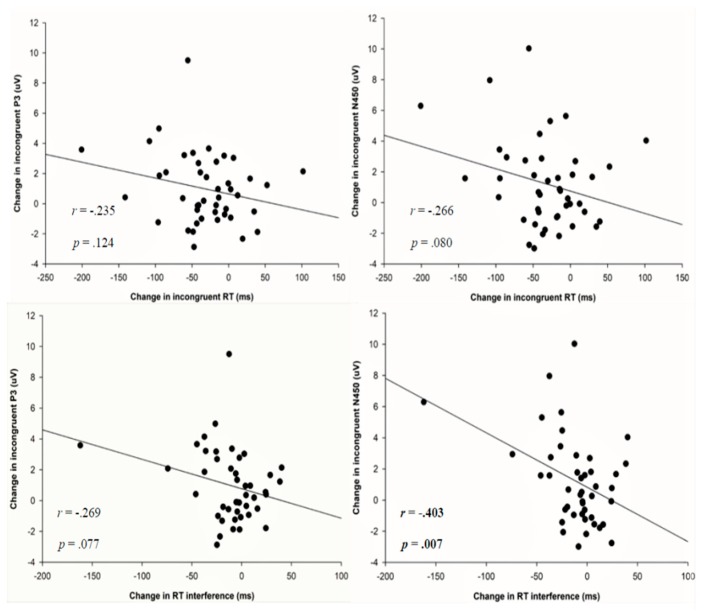
Scatter plots depict the bivariate correlations between changes in incongruent response time (RT) and changes in RT interference against changes in incongruent P3 amplitude and changes in incongruent N450 amplitude.

Based on the results of ANCOVAs, data on change in incongruent RT, change in RT interference, change in incongruent P3 amplitude, and change in incongruent N450 amplitude were selected for bivariate correlation analyses. Results only found significant correlations between changes in RT interference and changes in incongruent N450 amplitude (*r* = −0.403, *p* = 0.007). There were no significant correlations between the other measures (e.g., incongruent condition RT and incongruent P3, incongruent RT and incongruent N450, RT interference and incongruent P3). Figure 6 depicts these correlations.

## 4. Discussion

The current study focused on the examination of evidence supporting possible neural markers of improved cognitive inhibition following acute CAE in young and older adults, with particular interest in N450 and P3 amplitudes which reflect conflict monitoring and attention engagement processes. The current study is one of the first to explore the possible cognitive processes involved in cognitive inhibition following acute exercise, and is also the first to investigate the effects of acute exercise on N450 and P3 among older adults. Behaviorally, our finding indicated that acute CAE facilitated RT during Stroop performance, irrespective of task difficulty. With respect to neural markers, acute CAE decreased N450 regardless of task difficulty; bivariate correlations further revealed a significant correlation between change in RT interference and change in N450 amplitude in the incongruent trials following exercise, supporting a close relation between N450 and cognitive inhibition following exercise. Acute CAE also resulted in larger P3 irrespective of task demands, which replicated previous findings [13,17,30,31,32]. Lastly, similar size changes in N450 and P3 amplitudes were found for both young and older adults.

With respect to the effects of acute CAE on behavioral performance, the current findings showed that a single bout of CAE improved Stroop performance in task trails involving both smaller and larger cognitive demands as signified by shorter RT, with the benefits observed in both age groups. Similar acute CAE-induced improvements in inhibition-related tasks have been found in young [13,15,17,30,31] and older adults [14,16,32]. The current finding, together with others, suggests a general facilitation regarding the speed of information processing in association with inhibitory control. Importantly, this exercise-induced improvement in processing speed is unlikely to have resulted from a speed-accuracy tradeoff given the insignificant exercise effect on response accuracy. Moreover, RT interference scores were smaller following CAE compared to video-watching, providing additional support for improved inhibition performance.

However, our finding on response accuracy is inconsistent with Chang et al. [15] who found higher response accuracy following similar exercise protocol. This may be explained by our decision to adopt a longer response window (i.e., 1500 ms) than the Chang et al. study (i.e., 1000 ms) in order to include sufficient trials for older adults who tended to respond more slowly. As a consequence, the potential benefit of CAE on response accuracy may have been obscured due to a ceiling effect. This speculation is supported by the fact that response accuracy in our participants (i.e., approximately 98% and 91% for congruent and incongruent trials across age groups and conditions) were higher than young adults in the Chang et al. study (approximately 90% and 86% for congruent and incongruent trials). This difference in task design has highlighted the need for future studies to consider the specification of the length of the response window.

A novel aspect of the current study is its exploration of the role of N450 as one of the neural markers of improved cognitive inhibition following acute exercise. Results indicated smaller N450 amplitude following exercise regardless of congruency, which are in alignment with the behavioral finding of general facilitation, and are in agreement with a previous study [13]. Given N450 is an index of performance monitoring under competing response options [18], decreased N450 amplitude following CAE may imply a more efficient state of detection and monitoring of conflict evoked by competing representations [18]. Notably, our argument that N450 is one of the neural markers of improved cognitive inhibition is supported by a significant correlation between changes in RT interference and changes in N450 amplitude of incongruent trials following CAE. The current study, therefore, provides evidence that improved monitoring of conflict resulting from acute exercise mediates improved cognitive inhibition. The smaller N450 amplitude following acute exercise corroborates the finding of Li et al. [53] who demonstrated a deactivation in the ACC, the brain area responsible for detection of conflict in performance or environment, following exercise. Our findings also corroborate a recent study which suggested that the N2 component as an inhibition marker underlies improved Flanker performance [54], and extends that finding to different inhibition-related task (i.e., the Stroop test). We believe this is important given that the Flanker task involves different aspects of inhibition from the Stroop test. During the Flanker task, individuals are presented with an array of five arrows and are instructed to respond according to the direction of the central, target arrow while resisting interference from four flanking arrows pointing in the opposite direction. This task challenges the ability to overcome interference at a perceptual level (i.e., perceptual inhibition) [55]. On the other hand, while the difference in functional significance between N2 and N450 remains controversial, studies have suggested that, rather than a neural marker of conflict detection, N2 represents processes associated with the biasing of attention to the target stimulus, with larger amplitude associating with higher-probable stimulus [56,57]. Therefore, it was possible that the relationship between Flanker task performance and N2 may have been different from the relationship between Stroop test performance and N450. The finding of the current study has provided support for the idea that improved cognitive inhibition following acute exercise may be accounted for, at least in part, by a more efficient conflict monitoring signified by N450.

Regarding modulation in P3, our results have replicated previous findings. Participants had larger P3 amplitude following CAE relative to video-watching, regardless of congruency. This finding can be interpreted as acute CAE leading to a general facilitation of attention engagement regardless of task difficulty [29]. Such generalized effects of acute exercise can be interpreted in light of other work assessing perceptual inhibition [30,31,32] and motor inhibition [58]. Recently, a study from McMorris [59] has suggested that acute exercise could result in the secretion of neurotransmitters, such as norepinephrine (NE) and dopamine (DA), which increases vigilance and arousal, and are thought to be responsible for facilitation in tasks demanding attention control [60]. Furthermore, release of NE stimulates the firing of the noradrenergic receptors in the brain [61], which, in turn, strengthens neural signaling [62] and secretion of DA activates the dopaminergic receptors, thereby dampening neural noise [63]. It is possible that increased P3 amplitude following acute CAE are a result of the effect of NE and DA on vigilance, arousal, and neural reactivity.

It is also interesting to note that attention engagement and conflict monitoring, as signified by P3 and N450, may work synergistically to boost cognitive inhibition following acute exercise. Specifically, Silton et al. [20] suggests that, during the process of cognitive inhibition, successful implementation of top-down attention control interacts with the monitoring of conflicting response options. That is, in the context of there being sufficient attention engagement, reliance on later conflict monitoring decreases. Such cognitive modulations have recently been associated with up-regulation of proactive control [64]. Of course, more work is needed to explore the relationship between acute exercise, P3, and N450 in terms of cognitive inhibition. Collectively, our findings suggest that improved cognitive inhibition following acute exercise may be accounted for, at least in part, by modulations in the N450 inhibition marker and the P3 attention marker. While the findings on P3 have replicated previous research, those relating to N450 have made a novel contribution to the understanding of the cognitive underpinnings of behavioral performance.

Another novel aspect of the current study is the demonstration of N450- and P3-related cognitive gains in older adults, with the data indicating that older adults receive as much cognitive benefits as young adults from 20 min of aerobic exercise. This finding is relevant given that the elderly tend to experience declining conflict monitoring ability [23] and attention engagement [33]), which, in turn, may result in mind wandering [65], higher levels of distractibility [66], prolonged time required to implement correct responses [25], or frequent forgetting [26]. The finding in the current study of no age-related exercise-elicited differences is at odds with a recent meta-analysis [2], which concluded that there are smaller cognitive gains for young adults relative to older adults. This may be because only a relatively small number of the studies examined in the Ludyga et al. study reported data on older adults (7 as opposed to 23 studies involving young adults). The existence of only limited data increases the risk of bias from unrepresentative results. In fact, the current empirical evidence regarding age-related difference is somewhat contradictory. Dimitrova et al. [67] reported larger cognitive gains in older adults, but others found no such difference [68,69,70]. Nevertheless, it is hard to directly compares these studies since they differed in terms of whether they included a non-exercise condition (Dimitrova et al. did not), exercise modes (e.g., Hsieh et al. adopted resistance exercise whereas others adopted aerobic exercise), timing of cognitive assessments (e.g., Labelle et al. assessed in-exercise cognitive performance while the others assessed post-exercise performance), and cognitive tasks (e.g., while Kamijo et al. employed the Flanker task and Hsieh et al. used the working memory task, the Stroop test was used by others). Alternatively, it is also possible that the sample size of the current study (n = 44) may not have been sufficient to detect significant age-related differences (i.e., type 2 error).

There are a number of limitations to the current study. First, the sample did not include women because of methodological concerns. Thus, it is recommended to future study recruit women to gain a more comprehensive understanding to the acute CAE-elicited benefits. Secondly, our data cannot be generalized to older adults with cognitive impairments, such as those with mild cognitive impairment (MCI) or Alzheimer’s disease (AD), given individuals with MCI and AD may have different neuropathology [71] or functional differences in the P3 component [72] compared with individuals with normal cognitive aging. It is possible that individuals with MCI or AD would have different P3 modulations following exercise. Thirdly, the current study utilized a within-subjects post-experimental design (i.e., comparisons between post-exercise and post-rest). The lack of a data collection prior to each condition leaves open a question as to the directionality of exercise-induced changes in cognitive performance. For instance, some research has indicated that acute exercise may maintain, rather than improve, behavioral performance and attention engagement during cognitive tasks [50]. Although the use of a counterbalanced, within-subjects design should have reduced the effect of this limitation, future investigations may wish to consider employ a randomized, crossover, pre- versus post-intervention design. Fourth, the present study focuses on only two underlying cognitive inhibition processes (i.e., conflict monitoring and attention engagement), and its findings may not generalize to other cognitive inhibition processes (e.g., conflict resolution, response selection). Lastly, there is a possibility that exercise-induced muscle artifacts contaminated the ERP data. However, research has indicated that although greater magnitudes of high-frequency (15–30 Hz) noise related to muscle artifacts may occur during exercise, such exercise-related increases in neural oscillations seem to disappear in the 3 min following exercise [73]. Given that cognitive measures in this study were taken 15 min after the cessation of exercise, it is unlikely that this was a problem in the current study.

In conclusion, the current study has suggested that enhanced conflict monitoring, as signified by changes to N450, could be one of the underlying cognitive processes leading to improved Stroop performance following acute exercise. In addition, our results replicate the findings of previous studies in relation to the effect of acute exercise on P3. Similar magnitudes of N450 and P3 effects associated with acute exercise were found in both young and older adults. Acute exercise has been promoted as a means of counteracting increasing levels of sedentary behavior [74], particularly in older adults [75] who also suffer from deteriorating levels of attention and inhibition. The current findings are relevant in this regard in that they suggest that older adults receive as much cognitive benefit as young adults from a single bout of exercise. Investigation into the neuroelectric underpinnings may provide a basis for developing appropriate aerobic exercise interventions to foster brain health and functioning. Moreover, the practical significance of the current findings is strengthened by the fact that the exercise protocol was based on the ACSM recommendations for promoting health across the lifespan [44]. It is recommended that future studies recruit women and older adults with impaired cognition, consider stronger study design (e.g., randomized, crossover, pre- versus post-intervention design), and take measures of other domains of inhibition (e.g., motor, perceptual) to better understand the acute exercise-cognition relationship.

## Figures and Tables

**Table 1:** Summary of Demographic, Anthropometric, and Fitness Measures.

	Young Adults	Older Adults	
Measure	M (SD)	M (SD)	*t*-Tests
Age (years)	24.0 (3.1)	70.0 (3.3)	*p* < 0.001
BMI (kg/m^2^)	23.1 (2.6)	23.5 (2.1)	*p* = 0.632
Education (years)	15.4 (2.2)	16.3 (2.1)	*p* = 0.177
SES (scale)	3.4 (1.3)	4.1 (1.2)	*p* = 0.087
BDI (points)	4.5 (5.0)	3.7 (4.6)	*p* = 0.562
MMSE (points)	N/A	28.2 (1.6)	N/A
IPAQ (METs)	2761.5 (1710.7)	2397.6 (1469.7)	*p* = 0.458
Digit span (points)	39.7 (4.8)	28.8 (5.3)	*p* < 0.001
Digit span standard score (points)	13.2 (2.8)	12.3 (2.3)	*p* = 0.250
VO_2peak_ (mL·kg^−1^·min^−1^)	54.2 (8.9)	33.8 (2.5)	*p* < 0.001
VO_2peak_ percent rank (%)	64.3 (28.6)	63.3 (11.7)	*p* = 0.884

Note. BMI = body mass index. SES = socioeconomic status. BDI = Beck Depression Index. MMSE = Mini Mental State Examination. IPAQ = International Physical Activity Questionnaire.

**Table 2:** Summary Table of Mean (±1 SD) on Behavioral and event-related brain potentials (ERP) Indices.

		**Accuracy (%)**			**RT (ms)**		
		**Congruent**	**Incongruent**	**SI Effect**	**Congruent**	**Incongruent**	**SI Effect**
Young	Exercise	98.0 (1.7)	91.4 (7.8)	−6.6 (7.4)	419.7 (43.3)	483.0 (76.3)	63.3 (43.1)
	Video	97.6 (2.8)	91.5 (6.1)	−6.1 (5.5)	443.3 (52.7)	514.6 (84.6)	71.3 (41.2)
Older	Exercise	98.9 (1.1)	91.2 (7.5)	−7.6 (7.0)	592.9 (79.0)	749.6 (129.1)	156.7 (70.8)
	Video	98.7 (1.8)	90.5 (7.2)	−8.2 (6.3)	610.2 (84.3)	781.2 (134.1)	171.1 (83.3)
		**P3 Amplitude**			**N450 Amplitude**		
		**Congruent**	**Incongruent**		**Congruent**	**Incongruent**	
Young	Exercise	11.6 (4.5)	10.8 (5.0)		10.9 (5.4)	10.1 (5.9)	
	Video	10.4 (3.9)	9.5 (4.3)		9.5 (4.0)	8.6 (5.0)	
Older	Exercise	8.2 (4.4)	6.8 (4.5)		8.6 (5.9)	6.6 (5.8)	
	Video	7.9 (4.1)	6.1 (3.9)		7.8 (5.3)	5.6 (4.7)	

**Table 3:** Summary Table of ANOVA/ANCOVA on Behavioral and ERP Measures.

Measure and Effect	df	*F*	*p*	η^2^_p_
Mean accuracy				
Congruency	1, 41	4.6	=0.038	0.10
Mean RT				
Group	1, 41	78.2	<0.001	0.66
Congruency	1, 41	24.2	<0.001	0.37
Condition	1, 41	25.2	<0.001	0.38
Condition × Congruency	1, 41	4.6	=0.037	0.10
SI in RT				
Group	1, 42	29.9	<0.001	0.42
Condition	1, 42	5.0	=0.030	0.11
P3 mean amplitude				
Group	1, 42	7.1	=0.011	0.14
Congruency	1, 42	30.9	<0.001	0.42
Condition	1, 42	7.5	=0.009	0.15
N450 mean amplitude				
Congruency	1, 42	24.7	<0.001	0.37
Condition	1, 42	8.9	=0.005	0.17
Group × Congruency	1, 42	4.8	=0.035	0.10

Note. Only significant results were reported.
